# The Impact of a Tax on Sugar-Sweetened Beverages on Health and Health Care Costs: A Modelling Study

**DOI:** 10.1371/journal.pone.0151460

**Published:** 2016-04-13

**Authors:** J. Lennert Veerman, Gary Sacks, Nicole Antonopoulos, Jane Martin

**Affiliations:** 1 School of Public Health, The University of Queensland, Brisbane, Australia; 2 WHO Collaborating Centre for Obesity Prevention, Deakin University, Melbourne, Australia; 3 Obesity Policy Coalition/Cancer Council Victoria, Melbourne, Australia; Central Queensland University, AUSTRALIA

## Abstract

This paper aims to estimate the consequences of an additional 20% tax on sugar-sweetened beverages (SSBs) on health and health care expenditure. Participants were adult (aged > = 20) Australians alive in 2010, who were modelled over their remaining lifetime. We used lifetable-based epidemiological modelling to examine the potential impact of a 20% valoric tax on SSBs on total lifetime disability-adjusted life years (DALYs), incidence, prevalence, and mortality of obesity-related disease, and health care expenditure. Over the lifetime of adult Australian alive in 2010, seemingly modest estimated changes in average body mass as a result of the SSB tax translated to gains of 112,000 health-adjusted life years for men (95% uncertainty interval [UI]: 73,000–155,000) and 56,000 (95% UI: 36,000–76,000) for women, and a reduction in overall health care expenditure of AUD609 million (95% UI: 368 million– 870 million). The tax is estimated to reduce the number of new type 2 diabetes cases by approximately 800 per year. Twenty-five years after the introduction of the tax, there would be 4,400 fewer prevalent cases of heart disease and 1,100 fewer persons living with the consequences of stroke, and an estimated 1606 extra people would be alive as a result of the tax. The tax would generate an estimated AUD400 million in revenue each year. Governments should consider increasing the tax on sugared drinks. This would improve population health, reduce health care costs, as well as bring in direct revenue.

## Introduction

Unhealthy diets (11%) and high body mass index (9%) are the risk factors that contribute most to the burden of disease in Australia [1]. In order to reduce diet-related diseases, overweight, and obesity, focus should be placed on creating healthy food environments, whereby foods and beverages that contribute to a healthy diet are more readily available, affordable, and physically accessible, compared to unhealthy foods [2]. Food taxes have been frequently identified as a powerful tool to improve population diets [3], with evidence indicating that taxes are an effective intervention to improve the healthiness of consumption patterns [4]. The World Health Organization (WHO) recommends that country-level programs to combat obesity should include economic tools, such as taxes and subsidies, to improve the affordability of healthy food products and discourage the consumption of unhealthy options [2]. Several countries have enacted food taxes to improve population health, most notably Mexico, France, Hungary and a number of countries in the Western Pacific [5]. Sugar-sweetened beverages (SSBs) are the most commonly recommended target for food taxes, primarily due to the strong association with poor health and obesity [6], and their lack of nutrition and health benefits. In Australia, despite recommendations to consider increasing taxes for energy-dense foods (such as SSBs) [7], no such policies have yet been introduced.

The recently reviewed dietary guidelines clearly articulate the impact of SSBs on overweight and obesity in children and adults and offer recommendations to reduce consumption. Price can effectively influence the consumption of SSBs, with a price increase leading to reduced consumption [4]. Correspondingly, there is a growing body of evidence that suggests a tax on SSBs could reduce consumption and improve population weight and health outcomes, if the tax is sufficiently high [3]. UK-based research has confirmed the potential for an SSB tax to impact obesity rates, finding that a 20% tax on SSBs would lead to a reduction in the prevalence of obesity in the UK of 1.3% (approximately 180,000 people), with the greatest effects likely to be seen in young people, who are the highest consumers of SSBs [8]. Similarly, US-based research indicates the potential for substantial health gains from taxing SSBs in the US [9].

The limited available evidence on the economic impact of food taxes has been cited by policy makers as a major barrier to policy progress in the area of regulatory interventions such as taxes on unhealthy foods [10]. Accordingly, local data outlining the potential impact of taxing SSBs is particularly useful to policy-makers, with regards to government return on investment in relation to health sector costs.

The potential for food taxes to improve health outcomes in Australia has previously been examined. For example, Sharma et al recently examined the potential impact of a SSB tax in Australia, using household panel data [11]. They estimated that a 20% valoric tax (resulting in a percentage increase in price, as opposed to an excise tax that varies with the content of the product) could reduce total energy consumption by about 10,000 kJ per person per year, and body weight by 0.93 kg, at an average cost per household of $17. The effects were more pronounced among low income groups. However, previous studies are not based on the most recent dietary intake data for Australia and do not provide an indication of the magnitude, impact, and timing of health benefits resulting from the tax.

This paper aims to estimate the consequences of an additional 20% tax on SSBs in Australia on health and health care expenditure, using the latest dietary intake date for the Australian population.

## Methods

### Specification of the tax

We examined the potential impact of a 20% valoric tax on SSBs, simulating the impact if the SSB tax was in place from 2010 onward. An SSB was defined as a non-alcoholic drink with added sugar, including carbonated soft drinks and flavoured mineral waters. Fruit juices and drinks, energy drinks, milk-based drinks, and cordials were excluded. The tax was assumed to apply in addition to the existing Goods and Services Tax (GST), with the effect that the consumer price of SSBs would increase by 20%. This assumed that the tax would be fully passed on to the consumer.

### Effects of tax on body weight

Current dietary intake data was based on the Australian Health Survey (AHS) 2011–2013 [12]. In our base case analysis, we assumed that producers pass on the price increase in full to the consumers. A recent review of empirical evidence suggests that SSB taxes are passed on in full when they are applied to entire countries (but not necessarily when applied to smaller areas) [13]. In Mexico, there is evidence of overshifting in response to the SSB tax [14]. Australia-specific price elasticities were used to estimate how changes in price resulting from the tax would lead to changes in food purchases in the Australian adult population, based on the recent analysis by Sharma et al. [11]. Price elasticity indicates how price influences demand. An ‘own-price’ elasticity of -1 indicates that for every percent increase in price, demand drops by 1%, and a ‘cross-price elasticity’ indicates the change in demand for a product (e.g. milk) as a result of a price change of another product (e.g. SSBs). The own-price elasticity estimate for soft drinks (-0.63; p<0.01) was lower than global estimates (mean: -1.30; 95% CI: -1.09 to -1.51) from a recent systematic review and meta-analysis of studies in the USA, Mexico, Brazil, and France [15]. This lower value may reflect differences in food purchasing habits and culture in Australia, resulting in a more conservative estimate of the reduction in soft drink consumption in response to the tax. The only cross-price elasticity used, based on the statistically significant results in the Sharma et al study, related to artificially-sweetened soft drinks (0.16). Estimated changes in mean daily energy intake for each age and sex group were determined based on the estimated changes in purchases and the average energy density of relevant products [16]. Changes in quantity purchased were assumed to lead to changes in what was actually consumed, with no compensatory changes in physical activity levels. Resultant changes in body weight for each age and sex group were calculated using the ‘rule-of-thumb’ published by Hall et al (Lancet 2011) whereby every change in energy intake of 100 kJ per day results in an eventual body weight change of approximately 1kg [17]. This was converted to an estimated change in BMI for each age and sex group, based on mean heights and weights derived from the Australian Health Survey 2011/12 [12].

### Effects of body mass on health outcomes and health care costs

In order to estimate the effects of changes in BMI on health outcomes and health care costs we used a proportional multi-state life table model [18]. For each age-sex group, the consequences of weight change on the incidence of obesity-related diseases were calculated using potential impact fraction calculations and continuous risk functions (S1 Table, S2 Table).

The consequences on health and health care expenditure were modelled for all adult (aged > = 20) Australians alive in 2010, with life time follow up. BMI was modelled as lognormally distributed. Interventions shift the mean of those distributions. As in our earlier work in the ACE Prevention project, a trend towards higher mean BMI over time was assumed to last until 2023, after which BMI was assumed stable [19]. The model compares a scenario in which a tax on SSB is applied, with a business-as-usual scenario.

Nine obesity related diseases were modelled: stroke, ischemic heart disease, hypertensive heart disease, diabetes mellitus, osteoarthritis, post-menopausal breast cancer, colon cancer, endometrial cancer, and kidney cancer. Changes in disease incidence resulted in changes in prevalence at higher ages and later in time, and ultimately disease-specific mortality followed. Changes in disease-related quality of life at every age were calculated using disease-specific disability weights [19]. Disease-specific changes fed into a life table to calculate the number of health-adjusted life years lived (DALYs). To show the impact at the level of individuals, we also estimated the average lifetime impact for a hypothetical cohort of 20 to 24 year old Australians.

### Costs

To estimate the costs of legislative intervention we used WHO estimates for legislative change [20] and assumed 30 years of monitoring (Level 4 admin officer, annuitized with 3% discounting), as we did in previous work [18]. Health care costs by disease were those prepared for the ACE Prevention project [21]. The 2003 figures were inflated to 2010 values using national health price inflation estimates [22]. Health care costs for non-obesity related diseases were included in the model, so ‘unrelated’ health care costs in added years of life are accounted for [23].

### Implementation and sensitivity analysis

Calculations were performed in MS Excel (Microsoft Corporation, Redmond, Washington, USA) (S1 Model). Uncertainty was assessed by Monte Carlo simulation using the Ersatz program (Epigear.com, Brisbane, Australia; 2000 iterations), incorporating uncertainty in intervention effect on mean BMI, relative risks of incident disease and intervention costs (S1 Table, S2 Table). In this paper we report undiscounted results. We performed a one-way sensitivity analysis to explore the impact of the expected BMI trend, the duration of the effect on body mass, the pass-on rate, and discounting of future health gains and costs. Ethics approval was not required.

## Results

### Health outcomes

The 20% increase in price would result in an average change in consumption of SSBs from 141 g/day to 124 g/day across the Australian adult male population and from 76 to 67 g/day for women, representing a 12.6% decrease. Average energy intake would go down by 16 kJ/day for men and 9 kJ/day for women, and average body mass index of men by 0.10 kg/m2 (0.09–0.11), which is about 320g for 1.78m males. Women would lose 0.06 kg/m2 (0.06–0.07), or about 170g, assuming a height of 1.63m. The reductions in BMI would be larger for younger age groups (who consume higher volumes of SSBs) than older age groups. The tax could result in a decline in the prevalence of obesity of about 2.7% (0.7 percentage point) among men, and 1.2% (0.3 percentage point) among women, compared to business as usual.

**Fig 1:**
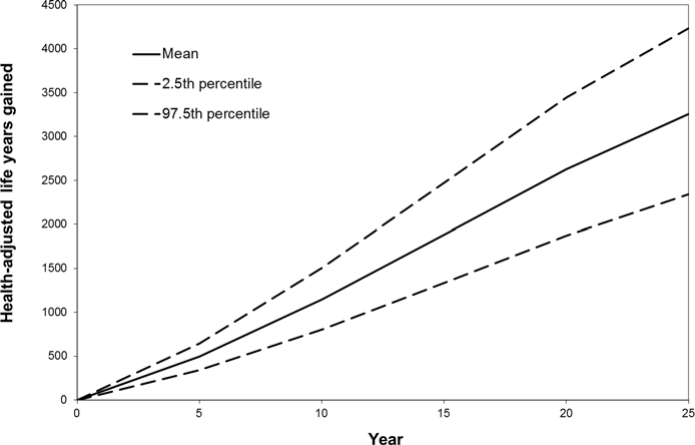
Annual number of health adjusted life years gained over time after implementation of a 20% tax on sugar sweetened drinks in Australia.

Over the lifetime of Australian adults alive in 2010, these seemingly modest changes in body mass translated to gains of 112,000 health-adjusted life years for men (95% uncertainty interval [UI]: 73,000–155,000) and 56,000 (95% UI: 36,000–76,000) for women. Fig 1 shows that the annual gains rise almost linearly over the first 25 years after the start of the tax. The estimated benefit for 20–24 year old males is the equivalent of about 7.6 days in full health, of which 4.9 in life extension and 2.7 in improved quality of life. For their female peers the model predicts 3.7 health-adjusted days gained, of which 2.2 from increased longevity.

**Fig 2:**
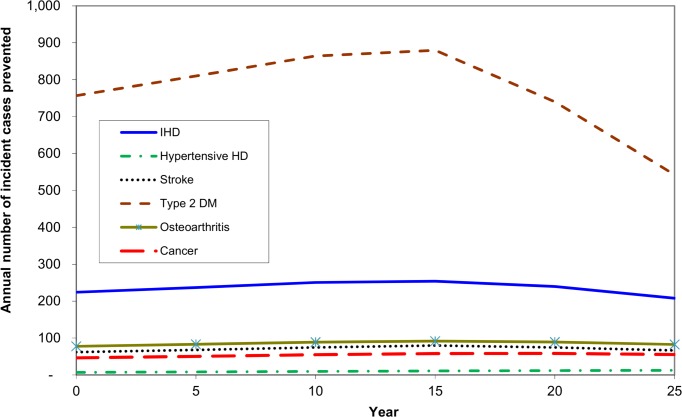
Projected annual number of new cases of disease prevented over time after implementation of a 20% tax on sugar sweetened drinks in Australia.

**Fig 3:**
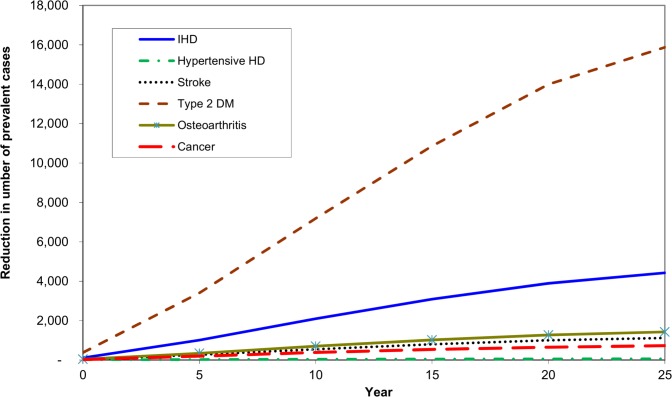
Projected number of existing (prevalent) cases of disease prevented over time after implementation of a 20% tax on sugar sweetened drinks in Australia.

**Fig 4:**
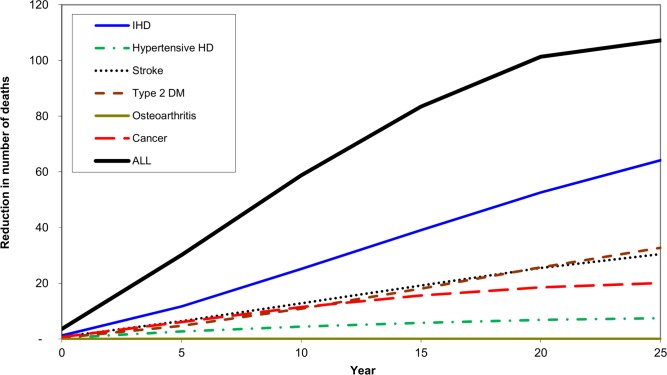
Projected number of deaths prevented over time after implementation of a 20% tax on sugar sweetened drinks in Australia, by cause of death.

Figs 2–4 show the results for the modelled diseases over the first 25 years after the introduction of the tax. (S3 Table of the Supporting Information presents more detail, including uncertainty intervals.) Most notably, the 20% tax would reduce the number of type 2 diabetes cases, with incidence down by approximately 800 per year. This is roughly a 0.6% reduction compared to the number expected without the tax (approximately 130,000 per year). The annual number of new cases of heart disease and stroke would be reduced by 240 and 70, respectively.

After 25 years there would be 16,000 less prevalent cases of diabetes, 4,400 fewer cases of IHD and 1,100 of stroke. In total, an estimated 1600 fewer deaths will occur by year 25, with heart disease accounting for the largest share of this postponed mortality.

### Costs

**Fig 5:**
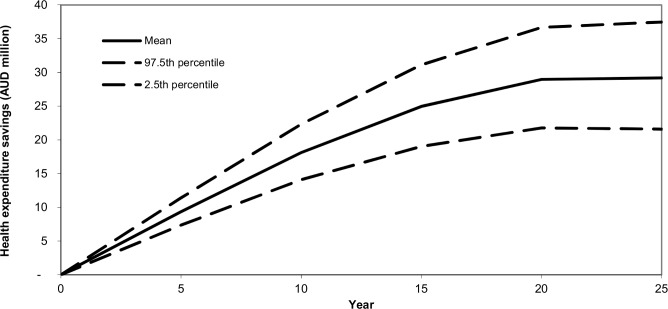
Health care cost savings over time after implementation of a 20% tax on sugared drinks in Australia.

The cost to government of implementing the tax was estimated at AUD27.6 million. The overall health care expenditure over the lifetime of the 2010 population aged > = 20 would be reduced by AUD609 million (95% UI: 368 million– 870 million). Fig 5 shows that the annual health care cost savings rise over the first 20 years and then stabilise at around AUD29 million per year.

### Revenue generated by the tax

In addition to the health care cost savings, the intervention would also raise substantial revenue for the government. Based on national household expenditure data in 2009/2010 [24], expenditure on soft drinks amounted to AUD5.32 per household per week, indicating that a 20% tax on these products would raise taxation revenue in excess of AUD400 million each year (taking into account changes in consumption in response to the tax). This revenue could be put towards health promotion activities, or used to subsidise healthy foods.

### Sensitivity analysis

Table 1 shows that if body mass across the ages were to remain as in 2010 rather than continue to increase until 2023, the lifetime health benefits would be 10% lower than projected in our base case scenario. Limiting the effect of the tax on body mass to the first 10 years reduces the health impact by 75%. The degree to which producers pass on the tax linearly relates to the size of the health benefits. Discounting future lifetime health gains by 3% has a relatively large impact, since the gains materialise over the course of several decades. The results for health care costs are in the same direction, but proportionally more modest in size. This is because health care costs are reduced in the near future, as costly cases of disease are prevented. After several decades, however, this is outweighed by more people remaining alive who would have died without the tax, and who accrue health care costs due to unrelated illness. In all scenarios, the policy was likely to be cost-saving from a health sector perspective.

**Table 1:** Results of the sensitivity analysis.

	Lifetime DALYs gained		Lifetime health care costs	
Base case	167,993		-$ 608,933,860	
Population BMI by age remains at 2010 levels	150,525	*-10%*	-$ 568,049,556	*-7%*
Effect of tax on BMI capped at 10 years	41,220	-75%	-$ 153,702,668	-75%
Tax pass-on 80%	134,865	*-20%*	-$ 484,016,283	*-21%*
Tax pass-on 120%	201,302	*+20%*	-$ 722,340,882	*+19%*
Health gain and costs discounted by 3%	63,167	*-62%*	-$ 423,214,932	*-30%*

## Discussion

Our analysis suggests that a 20% additional tax on SSBs would result in modest reductions in BMI and the proportion of Australians that are obese. This would nevertheless translate into health gains adding up to around 170,000 healthy life years over the lifetime of the 2010 Australian adult population. The costs of legislation and monitoring of the tax would be paid back around 14 times over in the form of reduced health care expenditure.

The weight loss we estimated is slightly less than that predicted by Sharma et al for a 20% valoric tax, which could be due to our more restricted definition of SSBs and/or the nature of the Australian Health survey data we used, where recall bias may have led to underestimation of the consumption of unhealthy foods [11]. The results in this study are also in line with our earlier findings that a broader ‘junk food’ tax (that included SSBs) could reduce mean weight by around 1.6 kg and avert 559,000 DALYs for the Australian adult population [18]. For comparison, individual diet and exercise interventions were estimated to avert up to 14,000 DALYs [19], and offering one year of orlistat (a weight-loss drug) treatment to all obese Australians was estimated to avert up to 8,800 DALYs [25].

Both empirical and modelled studies in various countries have shown that food taxes can improve diet, but few report to the level of health and economic outcomes [4]. The current study is the first Australian study to report results of the likely impact of a tax on SSBs by disease outcome and health care expenditure, including the timing that benefits are likely to accrue.

A strength of our analysis is that it presents outcomes at various levels, from changes in average BMI via a broad range of diseases, to healthy life years gained and health care expenditure changes over time. This is particularly important information for policy makers, for whom the timeframe for return on investment is likely to be an important consideration [10].

Another strength of the paper is that it uses Australian price elasticity estimates. However, unlike in the other literature [15] where consumption of milk, fruit juice, and artificially sweetened drinks were shown to be affected by changes in the price of SSBs, the only cross-price elasticity that was statistically significant for Australia related to artificially sweetened drinks [11]. If other food consumption compensation occurs in response to the tax, this would alter the results.

In this paper we did not consider the impact of a SSB tax on different socio-economic groups. Nevertheless, we note that Australians of low SES are disproportionately affected by high rates of diet-related illnesses [26] and are therefore likely to experience greater dietary improvements as a result of a tax on SSBs [27]. Inequitable aspects are likely to be further ameliorated if revenue was used to support healthy eating initiatives and subsidies on healthy foods for low-SES households. Detailed analysis of differential impacts by SES groups should be the focus of future research.

This paper investigated the impact of a valoric tax on SSBs. We note that there are a number of mechanisms that could be used to apply a tax on SSBs in the Australian context. Sharma et al has indicated that a volumetric tax (a tax on the quantity of sugar, which would most likely take the form of an excise tax in the Australian context) may have a greater impact on weight change and prove more efficient than a valoric tax. Our results are thus likely to be a conservative estimate of the health impact of a tax on SSBs in Australia.

## Conclusions

A multi-sectoral policy response is needed to address unhealthy diets and obesity in Australia and internationally. A tax on SSBs has the potential to reduce the burden of disease attributable to consumption of sugary drinks and the associated health care costs in the short term. It would also raise funds that can be used for a comprehensive strategy to improve diet and population health. While a tax on SSBs is not currently on the political agenda in Australia, drawing on this evidence and international experience, a tax on SSBs should be considered as part of Australia’s tax reform agenda.

## Supporting Information

S1 Model Obesity model SSB tax Australia PLoS ONE.xlsx. (XLSX)

S1 Table Input parameters (ex. relative risks). (PDF)

S2 Table Relative risks of disease per 1 unit increase of BMI. (PDF)

S3 Table Results of nine diseases over the first 25 years after the introduction of the tax. (PDF)
