# Supplementary material for: The Impact of a Tax on Sugar-Sweetened Beverages on Health and Health Care Costs: A Modelling Study
Source: PLoS One. 2016 Apr 13;11(4):e0151460. doi: 10.1371/journal.pone.0151460 (PMC4830445; doi:10.1371/journal.pone.0151460)
Supplement: S1 Table — (PDF) [file pone.0151460.s002.pdf]

The impact of a tax on sugar-sweetened beverages on health and health care costs: a modelling study – S1 Table

Table S1: Input parameters (ex. relative risks)

Parameter	***Value*** *Mean (SD)*	***Uncertainty distribution***	***Sources and assumptions***
Sugared drinks consumption	Varies by age and sex; see source	Normal	ABS Australian Health Survey 2011/12; Mean intake 'Soft drinks, and flavoured mineral waters' (g/day)
kJ / g of soft drink	1.8	None	CalorieKing.com.au
Own-price elasticity (regular soft drinks)	-0.63	None	Sharma et al (1)
Population numbers	See source	None	ABS 3101.0 Australian Demographic Statistics Table 9 – Estimated Resident Population
Overall mortality rates	See source	None	ABS 3302.0 - Deaths, Australia, 2010
Body mass index	See source	None	Predictions developed by Haby et al based on 11 cross-sectional national or state population surveys conducted in Australia between 1969 and 2004 (2)
Disease epidemiology	See source	None	Australian Burden of Disease 2003 with trends extrapolating to 2010 (3)
Relative risk (RR) of obesity related disease per BMI-unit increase	See Table S2	Normal (ln RR)	Relative risks by age from the World Health Organization’s Comparative Risk Assessment project, 2003 (4).
Health care expenditure	See source	None	Australian Institute of Health and Welfare Disease Costs and Impacts Study 2001 (5); inflated to 2010 values using AIHW health price deflators (6)
Cost of implementing, administering and enforcement of legislation (government)	20.3 ; 27.6 ; 34.8 million $	Triangular	Most likely value based on World Health Organization (WHO) estimate for Australia of cost of changing legislation regarding alcohol use (7). Other values = most likely estimate ±20%

References

1. Sharma A, Hauck K, Hollingsworth B, Siciliani L. The effects of taxing sugar-sweetened beverages across different income groups. Health economics. 2014;23(9):1159-84.
2. Haby MM, Markwick A, Peeters A, Shaw J, Vos T. Future predictions of body mass index and overweight prevalence in Australia, 2005-2025. Health Promot Int. 2012;27(2):250-60.
3. Vos T, Carter R, Barendregt J, Mihalopoulos C, Veerman JL, Magnus A, et al. Assessing Cost-Effectiveness in Prevention (ACE–Prevention): Final Report. Brisbane/Melbourne: University of Queensland, Brisbane and Deakin University, Melbourne., 2010.
4. Ezzati M, Lopez AD, Rogers A, Murray CJL. Comparative Quantification of Health Risks. Geneva: World Health Organisation; 2003.
5. Australian Institute of Health and Welfare. Disease costs and impacts study data. Canberra: Australian Institute of Health and Welfare, 2001.
6. Australian Institute of Health and Welfare. Health expenditure Australia 2009-10. Canberra: AIHW, 2011 Contract No.: Cat. no. HWE 55.
7. Chisholm D, Rehm J, Van Ommeren M, Monteiro M. Reducing the global burden of hazardous alcohol use: a comparative cost-effectiveness analysis. Journal of studies on alcohol. 2004;65(6):782-93.
