# Supplementary material for: The Impact of a Tax on Sugar-Sweetened Beverages on Health and Health Care Costs: A Modelling Study
Source: PLoS One. 2016 Apr 13;11(4):e0151460. doi: 10.1371/journal.pone.0151460 (PMC4830445; doi:10.1371/journal.pone.0151460)
Supplement: S2 Table — (PDF) [file pone.0151460.s003.pdf]

Table S2: Relative risks of disease per 1 unit increase of BMI (1, 2)

	Age	Males	Females
Colorectal cancer	<35	1	1
	35+	1.03 (1.01-1.05)	1.03 (1.01-1.05)
Breast cancer	<35	-	1
	35+	-	1.03 (1.02-1.04)
Endometrial cancer	<35	-	1.10 (1.07-1.14)
	35+	-	1.10 (1.07-1.14)
Kidney cancer	<35	1.06 (1.03-1.08)	1.06 (1.03-1.08)
	35+	1.06 (1.03-1.08)	1.06 (1.03-1.08)
Osteoarthritis	<35	1.04 (1.03-1.06)	1.04 (1.03-1.06)
	35+	1.04 (1.03-1.06)	1.04 (1.03-1.06)
Ischemic heart disease	<35	1	1
	35-44	1.12 (1.05-1.19)	1.12 (1.05-1.19)
	45-59	1.10 (1.08-1.14)	1.10 (1.08-1.14)
	60-69	1.06 (1.03-1.08)	1.06 (1.03-1.08)
	70-79	1.04 (1.02-1.06)	1.04 (1.02-1.06)
	80+	1.02 (1.00-1.05)	1.02 (1.00-1.05)
Hypertensive heart disease	<45	1	1
	45-59	1.09 (1.03-1.14)	1.09 (1.03-1.14)
	60-69	1.16 (1.05-1.27)	1.16 (1.05-1.27)
	70-79	1.12 (1.04-1.21)	1.12 (1.04-1.21)
	80+	1.06 (1.02-1.11)	1.06 (1.02-1.11)
Stroke	<35	1	1
	35-44	1.14 (1.05-1.23)	1.14 (1.05-1.23)
	45-59	1.10 (1.03-1.16)	1.10 (1.03-1.16)
	60-69	1.08 (1.03-1.13)	1.08 (1.03-1.13)
	70-79	1.05 (1.02-1.09)	1.05 (1.02-1.09)
	80+	1.03 (1.01-1.05)	1.03 (1.01-1.05)
Type II Diabetes	<35	1	1
	35-44	1.19 (1.06-1.32)	1.19 (1.06-1.32)
	45-69	1.14 (1.05-1.23)	1.14 (1.05-1.23)
	70+	1.10 (1.03-1.16)	1.10 (1.03-1.16)
NB. Values shown are the mean and 95% confidence intervals.			

References

1. Ni Mhurchu C, Parag V, Nakamura M, Patel A, Rodgers A, Lam TH. Body mass index and risk of diabetes mellitus in the Asia-Pacific region. Asia Pac J Clin Nutr. 2006;15(2):127-33.
2. James WP, Jackson-Leach R, Ni Mhurchu C, Kalamara E, Shayeghi M, Rigby NJ, et al. Overweight and obesity (high body mass index). In: Ezzati M, Lopez AD, Rodgers A, Murray CJL, editors. Comparative quantification of health risks. 1. Geneva: World Health Organization; 2004. p. 497-596.
