# Supplementary material for: The Impact of a Tax on Sugar-Sweetened Beverages on Health and Health Care Costs: A Modelling Study
Source: PLoS One. 2016 Apr 13;11(4):e0151460. doi: 10.1371/journal.pone.0151460 (PMC4830445; doi:10.1371/journal.pone.0151460)
Supplement: S3 Table — (PDF) [file pone.0151460.s004.pdf]

Table S3. Results of nine diseases over the first 25 years after the introduction of the tax

Expected numbers (with 95% uncertainty ranges) of incident cases, prevalent cases and deaths averted in the first to 25th year after implementation of a 20% tax on sugar-sweetened beverages in Australia. For incidence and mortality, numbers are for the stated year. For prevalence, they refer to the end of that year (e.g. Year 1 refers to prevalent numbers at 1 year after the tax change took effect).

	Ischaemic health disease	Hypertensive heart disease	Stroke	Type 2 diabetes mellitus	Osteo-arthritis	Colorectal cancer	Breast cancer	Endometrial cancer	Kidney cancer	Any cause
**INCIDENCE**										
Year 1	224	7	62	757	78	17	16	5	8	
	(157-295)	(4-11)	(31-93)	(379-1126)	(56-100)	(8-27)	(10-21)	(3-7)	(6-11)	
Year 5	237	8	68	810	83	19	17	6	9	
	(163-316)	(4-13)	(33-105)	(394-1245)	(60-106)	(9-30)	(11-23)	(4-8)	(6-12)	
Year 10	251	9	75	864	89	21	18	7	10	
	(171-339)	(5-15)	(36-118)	(410-1369)	(63-115)	(10-32)	(12-24)	(4-10)	(7-14)	
Year 15	254	11	80	880	92	22	18	7	11	
	(170-348)	(5-18)	(38-128)	(411-1413)	(65-120)	(10-34)	(12-25)	(4-11)	(7-15)	
Year 20	240	12	75	740	89	22	18	8	11	
	(159-329)	(6-20)	(34-120)	(348-1175)	(62-117)	(9-35)	(11-24)	(5-11)	(7-16)	
Year 25	208	13	66	542	83	21	16	7	11	
	(137-285)	(6-21)	(29-109)	(258-841)	(56-110)	(8-34)	(10-22)	(4-11)	(7-16)	
Cumulative 0-25	6,350	269	1,895	20,088	2,227	527	441	172	262	
	(4312-8591)	(135-436)	(897-3016)	(9671-31487)	(1578-2893)	(237-834)	(290-598)	(105-250)	(171-365)	
**PREVALENCE**										
Year 1	111	3	30	378	39	8	8	2	4	
	(78-146)	(2-5)	(15-46)	(189-562)	(28-50)	(4-13)	(5-10)	(2-3)	(3-5)	
Year 5	1,009	23	268	3,400	342	70	67	22	34	
	(703-1331)	(12-35)	(133-408)	(1679-5123)	(247-444)	(33-108)	(44-90)	(14-31)	(23-47)	
Year 10	2,095	37	546	7,191	701	129	136	48	67	

	(1457-2780)	(20-59)	(266-845)	(3485-11030)	(501-908)	(60-202)	(89-184)	(30-67)	(44-92)	
Year 15	3,082	48	798	10,861	1,020	172	197	73	94	
	(2141-4103)	(24-77)	(383-1258)	(5176-16879)	(719-1332)	(77-271)	(128-267)	(45-104)	(61-130)	
Year 20	3,891	57	1,002	13,999	1,271	198	246	96	114	
	(2688-5215)	(28-94)	(473-1605)	(6646-22016)	(886-1672)	(84-319)	(159-337)	(58-139)	(74-159)	
Year 25	4,426	62	1,118	15,875	1,427	208	279	113	126	
	(3047-5939)	(29-105)	(521-1809)	(7480-25050)	(979-1896)	(82-342)	(176-385)	(68-166)	(81-177)	
**MORTALITY**										
Year 1	1	0	1	0	0	0	0	0	6	4
	(1-2)	(0-1)	(0-1)	(0-1)	(0-0)	(0-1)	(0-0)	(0-0)	(4-9)	(3-4)
Year 5	12	3	6	5	0	3	1	0	7	30
	(7-16)	(1-4)	(3-10)	(2-7)	(0-0)	(2-5)	(1-2)	(0-0)	(4-10)	(24-37)
Year 10	25	4	13	11	0	6	2	1	7	59
	(16-35)	(2-7)	(6-20)	(5-17)	(0-0)	(3-9)	(2-3)	(0-1)	(4-11)	(46-73)
Year 15	39	6	19	18	0	8	3	1	8	83
	(25-54)	(3-9)	(9-30)	(8-28)	(0-0)	(4-13)	(2-5)	(1-1)	(5-12)	(65-103)
Year 20	53	7	26	26	0	9	4	1	8	101
	(34-74)	(3-11)	(11-41)	(12-41)	(0-0)	(4-15)	(3-6)	(1-2)	(5-13)	(79-126)
Year 25	64	7	30	33	0	10	4	1	8	107
	(41-90)	(4-13)	(13-50)	(15-52)	(0-0)	(4-16)	(3-6)	(1-2)	(4-12)	(83-133)
Cumulative 0-25	839	125	419	396	1	162	69	20	195	1,606
	(535-1173)	(63-202)	(192-668)	(182-625)	(0-1)	(70-258)	(45-94)	(12-28)	(112-292)	(1252-1986)
